# Diffusive mediator feedbacks control the health-to-disease transition of skin inflammation

**DOI:** 10.1371/journal.pcbi.1011693

**Published:** 2024-01-18

**Authors:** Maki Sudo, Koichi Fujimoto

**Affiliations:** 1 Department of Biological Sciences, Osaka University, Machikaneyama-cho, Toyonaka, Japan; 2 Program of Mathematical and Life Sciences, Graduate School of Integrated Sciences for Life, Hiroshima University, Kagamiyama, Higashi-Hiroshima, Japan; University of Oxford, UNITED KINGDOM

## Abstract

The spatiotemporal dynamics of inflammation provide vital insights into the understanding of skin inflammation. Skin inflammation primarily depends on the regulatory feedback between pro- and anti-inflammatory mediators. Healthy skin exhibits fading erythema. In contrast, diseased skin exhibits expanding erythema with diverse patterns, which are clinically classified into five types: circular, annular, arcuate, gyrate, and polycyclic. Inflammatory diseases with expanding erythema are speculated to result from the overproduction of pro-inflammatory mediators. However, the mechanism by which feedback selectively drives the transition from a healthy fading erythema to each of the five types of diseased expanding erythema remains unclear. This study theoretically elucidates the imbalanced production between pro- and anti-inflammatory mediators and prospective treatment strategies for each expanding pattern. Our literature survey showed that eleven diseases exhibit some of the five expanding erythema, thereby suggesting a common spatiotemporal regulation underlying different patterns and diseases. Accordingly, a reaction-diffusion model incorporating mediator feedback reproduced the five observed types of diseased expanding and healthy fading patterns. Importantly, the fading pattern transitioned to the arcuate, gyrate, and polycyclic patterns when the productions of anti-inflammatory and pro-inflammatory mediators were lower and higher, respectively than in the healthy condition. Further depletion of anti-inflammatory mediators caused a circular pattern, whereas further overproduction of pro-inflammatory mediators caused an annular pattern. Mechanistically, the bistability due to stabilization of the diseased state exhibits circular and annular patterns, whereas the excitability exhibits the gyrate, polycyclic, arcuate, and fading patterns as the threshold of pro-inflammatory mediator concentration relative to the healthy state increases. These dynamic regulations of diffusive mediator feedback provide effective treatment strategies for mediator production wherein skins recover from each expanding pattern toward a fading pattern. Thus, these strategies can estimate disease severity and risk based on erythema patterns, paving the way for developing noninvasive and personalized treatments for inflammatory skin diseases.

## Author summary

The spatiotemporal dynamics of inflammation provide vital insights into understanding inflammation. Healthy skin exhibits fading inflammation, while diseased skin exhibits expanding inflammation with circular, annular, arcuate, gyrate, and polycyclic patterns. Diseases with expanding inflammation are thought to be linked to an overproduction of pro-inflammatory mediators, but how does the mediator production selectively transition from healthy fading inflammation to five diseased expanding inflammation? We used a mathematical model incorporating production and diffusion of mediators in the skin to simulate the fading and expanding patterns. The model reproduced the five observed types of diseased expanding and healthy fading patterns. We found that the fading pattern transitions in the order of arcuate, polycyclic, gyrate, annular, and circular patterns with increasing production of pro-inflammatory mediators or decreasing production of anti-inflammatory mediators. Importantly, dynamic feedback between pro-inflammatory and anti-inflammatory mediators is responsible for their production balance and controls health-to-disease transition of skin inflammation pattern. This mechanism guides treatment strategies and helps skin recover from expanding patterns towards a fading pattern. This research can help estimate disease severity and risk based on inflammation patterns and has potential applications in noninvasive and personalized treatments for inflammatory skin diseases.

## Introduction

Spatiotemporal dynamics provide valuable insights into variability in inflammation. Normal inflammatory response occurs only in the affected area and subsides within a short period of time, whereas chronic inflammatory response expands to adjacent healthy tissue and persists for months or years [1]. Chronic inflammation is primarily attributed to an imbalance between pro- and anti-inflammatory mediators [2–4]. Hence, the prevention and treatment of chronic inflammation are required to elucidate the mechanisms of the imbalance involved.

**Fig 1:**
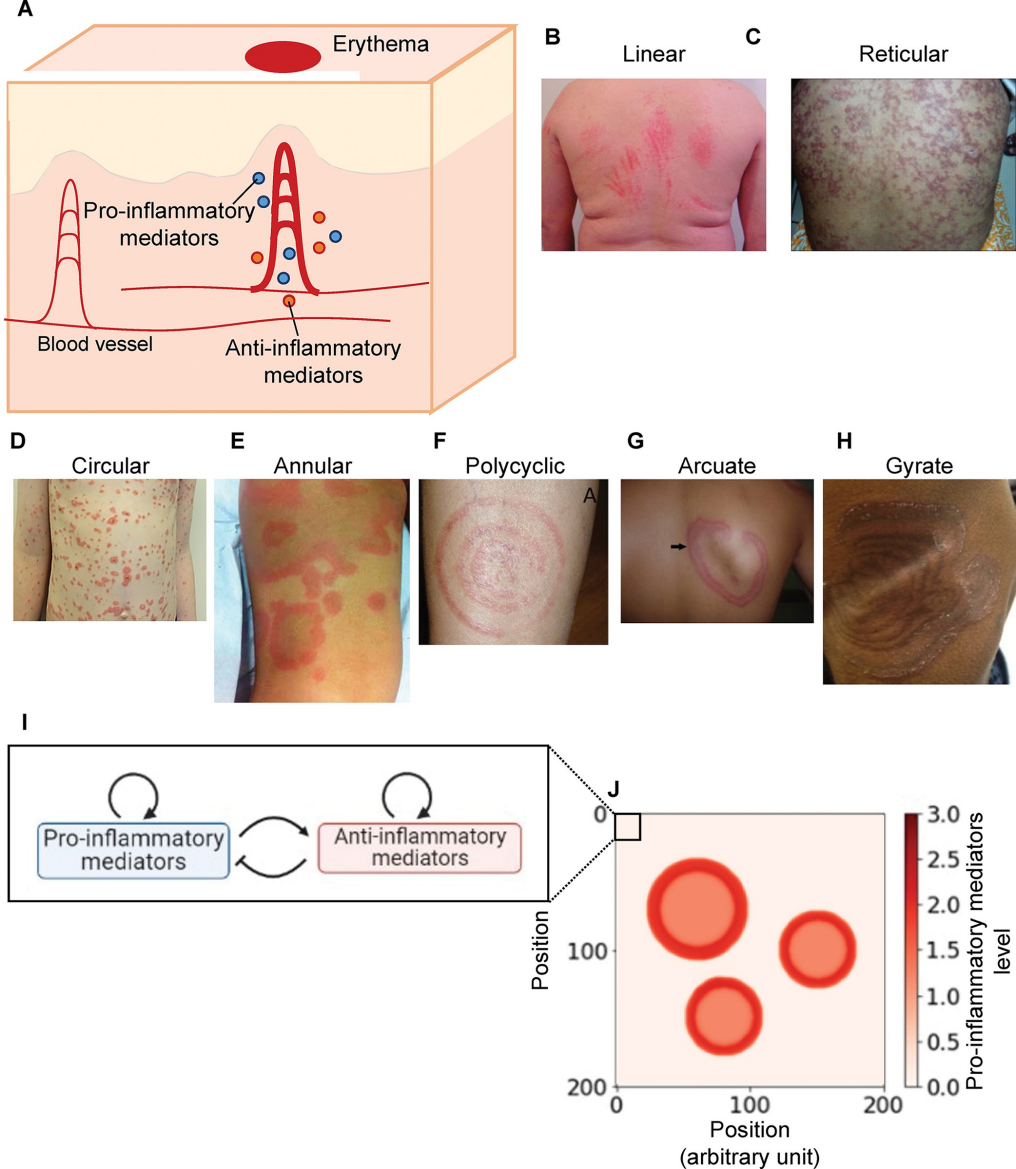
Erythema pattern and modeling of erythema development. **(A)** Process of the inflammatory response for erythema development. Upon stimulation, keratinocytes and resident immune cells secrete pro-inflammatory mediators that induce the production of pro- and anti-inflammatory mediators. Pro-inflammatory mediators dilate local blood vessels. The dilation appears as redness on the skin surface, developing erythema. **(B–H)** Photographs of erythema with linear [24] (B), reticular [25] (C), circular [26] (D), annular [27] (E), polycyclic [28] (F), arcuate [29](G), or gyrate patterns [30] (H)**. (I)** A model for regulatory feedback between pro- and anti-inflammatory mediators. **(J)** A representation of simulation in the skin. The skin surface is partitioned into square regions. Erythema is initiated by keratinocytes and immune cells in the skin through secreting pro-inflammatory mediators. The area of microinflammation with a high concentration of pro-inflammatory mediators is considered as a “seed” region, and its projection to the surface is colored in red.

The possibility for direct observation makes the skin an ideal system for studying the spatiotemporal dynamics of inflammation. Skin inflammation typically manifests as redness on the skin surface and is medically referred to as erythema [5]. Erythema appears when pro-inflammatory mediators (e.g., tumor necrosis factor [TNF]-α and interleukin [IL]-1) induce vasodilation and hyperemia in the dermis (Fig 1A). The production of pro-inflammatory mediators is influenced by characteristics of the skin, such as the skin barrier and microbiome [6,7]. Pro-inflammatory mediators induce the production of anti-inflammatory mediators (e.g., IL-4, IL-10, and IL-13), which reduce the production of the pro-inflammatory mediators as a regulatory feedback mechanism [2,8]. In addition to negative feedback, pro- and anti-inflammatory mediators induce their own production via positive feedback [2,9]. Experimental studies have revealed that dysregulation of feedback causes the overproduction of pro-inflammatory mediators and the transition from normal to chronic inflammation [2–4]. Normal inflammation in healthy skin appears as fading erythema, where redness decreases and eventually disappears [10]. Fading erythema includes a linear pattern reflecting the affected areas in contact with, for instance, harmful animal tentacles or plant branches, and a reticular pattern reflecting the capillary structure (Fig 1B and 1C) [10]. Erythema patterns in diseased skin differ from those in healthy skin: chronic inflammation in diseased skin appears as expanding erythema with circular, annular, polycyclic, arcuate, or gyrate patterns (Fig 1D–1H) [11]. Erythema expands for hours or days, with multiple expanding erythema leading to fusion [5,11]. Erythema patterns provide the first clue for the diagnosis and treatment of inflammatory skin diseases regulated by mediator feedback.

Dermatologists have reported numerous clinical findings on inflammatory skin diseases to identify appropriate treatment strategies. Clinical reports typically show the same erythema pattern in multiple diseases. For example, the annular pattern is common in erythema migrans, erythema multiforme, lichen planus, pityriasis rosea, psoriasis, tinea corporis, and urticaria [12]. Furthermore, a clinical report comparing patients with lyme disease revealed multiple patterns in the same disease; skin lesions in Missouri cases were more likely to show central clearing such as an annular pattern, whereas those in New York cases were more likely to show a circular pattern [13]. Moreover, three months after treatment, some New York patients remained fatigued or had joint pain, while Missouri patients did not have any of these prognostic symptoms, thereby suggesting a correlation between erythema patterns and treatment efficacy. As these inflammatory diseases primarily result from the overproduction of pro-inflammatory mediators, mediator production can affect the development of the expanding pattern observed in different diseases. Thus, elucidating which alterations in mediator production result in specific expanding erythema patterns across diseases will enable the estimation of fundamental treatment strategies.

Mathematical modeling has recently attracted attention for predicting treatment strategies for inflammatory skin diseases. A mathematical model incorporating regulatory feedback between pro- and anti-inflammatory mediators predicts the temporal dynamics of normal and chronic inflammation [14]. The model characterized normal inflammation as a system with one stable steady state, where mediator concentrations transiently increased upon stimulation and subsequently returned to their original levels, showing excitability. Alternatively, chronic inflammation is characterized as a system with additional steady states with persistently high or oscillating mediator concentrations. Although the model predicted a different number of steady states underlying the temporal dynamics between normal and chronic inflammation, the absence of mediator diffusion failed to account for spatial dynamics.

Mathematical models incorporating diffusion, referred to as reaction-diffusion models, have studied the spatial dynamics of erythema patterns [15–20]. A reaction-diffusion model for erythema gyratum repens suggested that the gyrate pattern characteristic of the disease is formed in the presence of excitability, where perturbations induce a transient response that returns to a stable steady state [16]. Other reaction-diffusion models for psoriasis and urticaria have shown that positive and negative feedback of pro-inflammatory mediators plays a major role in generating several expanding patterns including circular, annular, arcuate, and gyrate patterns [19,20]. These two models suggest that different expanding patterns within a single disease arise from alterations in mediator production due to slight differences in regulatory feedback strength. The psoriasis model also showed that the patterns faded after treatment by increasing the degradation rate of pro-inflammatory mediators. These studies focused on pro-inflammatory mediators rather than anti-inflammatory mediators. Another reaction-diffusion model incorporating pro- and anti-inflammatory mediators and chemotactic cells reproduced an expanding circular pattern [17]. Previous reaction-diffusion models, including chemotactic cells, have reproduced the resolution of inflammation in the lung [21,22]; however, the resolution of erythema has not received much attention. Although the overproduction of pro-inflammatory mediators is thought to cause expanding erythema in many modeling studies of these inflammatory skin diseases [15–20,23], the mechanism by which overproduction through the feedback selectively drives the transition from a healthy state with fading erythema to a disease state with each of the five types of expanding erythema remains unclear.

Elucidating this mechanism requires the development of a reaction-diffusion model for fading patterns in healthy skin. Improving the model to reproduce both fading and expanding erythema will provide a better understanding of the healthy-to-disease transition and suggest noninvasive treatment strategies. Moreover, developing a model that comprehensively reproduces all five types of expanding patterns in a disease-independent manner enables us to infer how the direction and severity of the mediator imbalance affect the clinical erythema pattern.

This study aimed to theoretically elucidate how the imbalance in the production of pro- and anti-inflammatory mediators causes each expanding pattern in multiple diseases and how to restore balance and return to the fading pattern. To this end, we examined whether the expanding patterns in multiple diseases result from the reaction-diffusion system with regulatory feedback (Fig 1A, 1I and 1J). Using the reaction-diffusion model, we explored the conditions of appearance and effective treatment strategies for each expanding pattern.

## Methods

### Source of information on the erythema patterns

First, clinical reports of erythema in the literature were reviewed to examine the association between erythema patterns and skin diseases. Expanding patterns were observed in eleven different diseases, including psoriasis, lupus erythematosus, bullous pemphigoid, lyme disease, erythema multiforme, lymphoma, annular erythema, sjögren’s syndrome, sweet syndrome, nummular eczema, and erythema gyratum repens [10]. We collected clinical photographs of erythema observed in patients whose photographs were extracted from clinical studies using literature searches in PubMed. For example, photographs of psoriasis have been reviewed using the “(psoriasis AND clinical AND pattern) OR (psoriasis AND clinical AND shapes) OR (psoriasis AND clinical spectrum)” search phrases. After reviewing the titles and abstracts, 132 relevant papers with clinical photographs were selected.

### Development of the reaction-diffusion model

A reaction-diffusion model was developed to investigate whether regulatory feedback and diffusion of pro- and anti-inflammatory mediators can generate erythema patterns. As pro-inflammatory mediators induce erythema through vasodilation, we used the concentration of pro-inflammatory mediators as an indicator of erythema. The variables of the model reflect the concentrations of pro-inflammatory mediators (*A*) and anti-inflammatory mediators (*I*). Pro-inflammatory mediators are present at low levels in the unstimulated skin through basal secretion [6]. In response to stimulation, keratinocytes and immune cells in the skin secrete pro-inflammatory mediators, which induce their production through positive feedback [9,31]. Pro-inflammatory mediators also induce the production of anti-inflammatory mediators through negative feedback [2,8]. The positive and negative feedback between pro- and anti-inflammatory mediators is shown schematically in Fig 1I. The production rate of pro-inflammatory mediators is biologically limited; therefore, the model function of *A* saturates the Hill function with the Hill coefficient representing cooperativity in the regulation, *n*. Pro-inflammatory mediators are assumed to degrade naturally at a constant rate [32]. To model these processes, the production of pro-inflammatory mediators (*A*) is represented by the autoregulation of *A* and repression by *I*:

$$\frac{\partial A}{\partial T}=P_{A}+\frac{{Q_{A}A}^{n}K_{I}}{{(A}^{n}+{K_{A}}^{n})(I+K_{I})}-R_{A}A+D_{A}\Delta A$$ (1A)

The first term *P*_*A*_ represents the basal production rate. The term $\frac{{Q_{A}A}^{n}}{A^{n}+{K_{A}}^{n}}$ captures the positive feedback, where *Q*_*A*_ and *K*_*A*_ are the maximum production rate and threshold of production of pro-inflammatory mediators, respectively. The term $\frac{K_{I}}{I+K_{I}}$ modulates the inhibitory effects of anti-inflammatory mediators. The third and fourth terms represent the degradation with *R*_*A*_ and diffusion *D*_*A*_ with *Δ* denoting the Laplacian operator ($\frac{\partial^{2}}{\partial x^{2}}+\frac{\partial^{2}}{\partial y^{2}}$), respectively.

Anti-inflammatory mediators induce their production through positive feedback [2,33]. Anti-inflammatory mediators are assumed to be present at low levels in the skin through basal secretion and naturally degrade at a constant rate. To model these processes, the production of anti-inflammatory mediators (*I*) was modeled as follows:

$$\frac{\partial I}{\partial T}=P_{I}+\frac{Q_{I}A^{n}I^{n}}{{(A}^{n}+{K_{A}}^{n}){(I}^{n}+{K_{I}}^{n})}-R_{I}I+D_{I}\Delta I$$ (1B)

The first, third, and fourth terms in Eq 1B represent the basal secretion, degradation, and diffusion of anti-inflammatory mediators at *P*_*I*_, *R*_*I*_, and *D*_*I*_, respectively. The second term of Eq 1B represents the induction of anti-inflammatory mediators by pro-inflammatory mediators and via the positive feedback of anti-inflammatory mediators, where *Q*_*I*_ denotes the maximum production rate of anti-inflammatory mediators.

The values of these parameters depend on the skin conditions. For example, experiments have suggested that the maximum production rate (*Q*_*A*_) of one type of pro-inflammatory mediator, IL-1*β*, increases with the deterioration of the skin microbiome [7] and that the basal secretion rate (*P*_*A*_) of IL-1*β* increases with a defect in skin barrier integrity [6]. Due to the lack of sufficient quantitative information on the kinetic parameter values and diffusion coefficients, we investigated the model dynamics for a wide range of parameters. For this purpose, the model was nondimensionalized using the following scaling:

$$A=K_{A}a,\;I=K_{I}i,\;T=\frac{t}{R_{I}},$$

where time is scaled with the degradation rate of anti-inflammatory mediators, which is expected to be in the order of minutes [34].

The final system of partial differential equations for pro- and anti-inflammatory mediators is given by:

$$\frac{\partial a}{\partial t}=p_{a}+\frac{q_{a}a^{n}}{{(a}^{n}+1)(i+1)}-r_{a}a+D_{a}\Delta a$$ (2A)

$$\frac{\partial i}{\partial t}=p_{i}+\frac{q_{i}a^{n}i^{n}}{{(a}^{n}+1){(i}^{n}+1)}-i+D_{i}\Delta i$$ (2B)

$$where\;p_{a}=\frac{P_{A}}{R_{I}K_{A}},q_{a}=\frac{Q_{A}}{R_{I}K_{A}},r_{a}=\frac{R_{A}}{R_{I}},D_{a}=\frac{D_{A}}{R_{I}},p_{i}=\frac{P_{I}}{R_{I}K_{I}},q_{i}=\frac{Q_{i}}{R_{I}K_{I}},D_{i}=\frac{D_{I}}{R_{I}}.$$

The meanings of these new parameters are summarized in Table 1. We set the Hill coefficient *n* to 2 to introduce the simplest form of cooperativity. The reaction terms of this model are similar to the classical activator-inhibitor model proposed by Turing [35], which includes the negative feedback of the activator through the inhibitor and the positive feedback of the activator. These reaction terms potentially result in Turing instability. However, the present model setting does not show Turing instability. The reason is that Turing instability requires a large difference between the diffusion coefficients of the activator and inhibitor [36], whereas these coefficients in the present model were set to be equal based on molecular findings that these molecular weights are close in proximity [3]. Hence, a reaction-diffusion equation consisting of pro- and anti-inflammatory mediators was used to simulate the development of erythema patterns.

**Table 1:** System parameters and their interpretations.

Parameter	Description
*p*_*a*_	Basal production rate for pro-inflammatory mediators
*q*_*a*_	Maximum production rate of pro-inflammatory mediators
*r*_*a*_	Relative rate of clearance of pro-inflammatory mediators to anti-inflammatory mediators
*D*_*a*_	Diffusion coefficient of pro-inflammatory mediators
*p*_*i*_	Basal production rate for anti-inflammatory mediators
*q*_*i*_	Maximum production rate of anti-inflammatory mediators
*D*_*i*_	Diffusion coefficient of anti-inflammatory mediators
*n*	Hill coefficient

### Numerical simulation of the model

**Fig 2:**
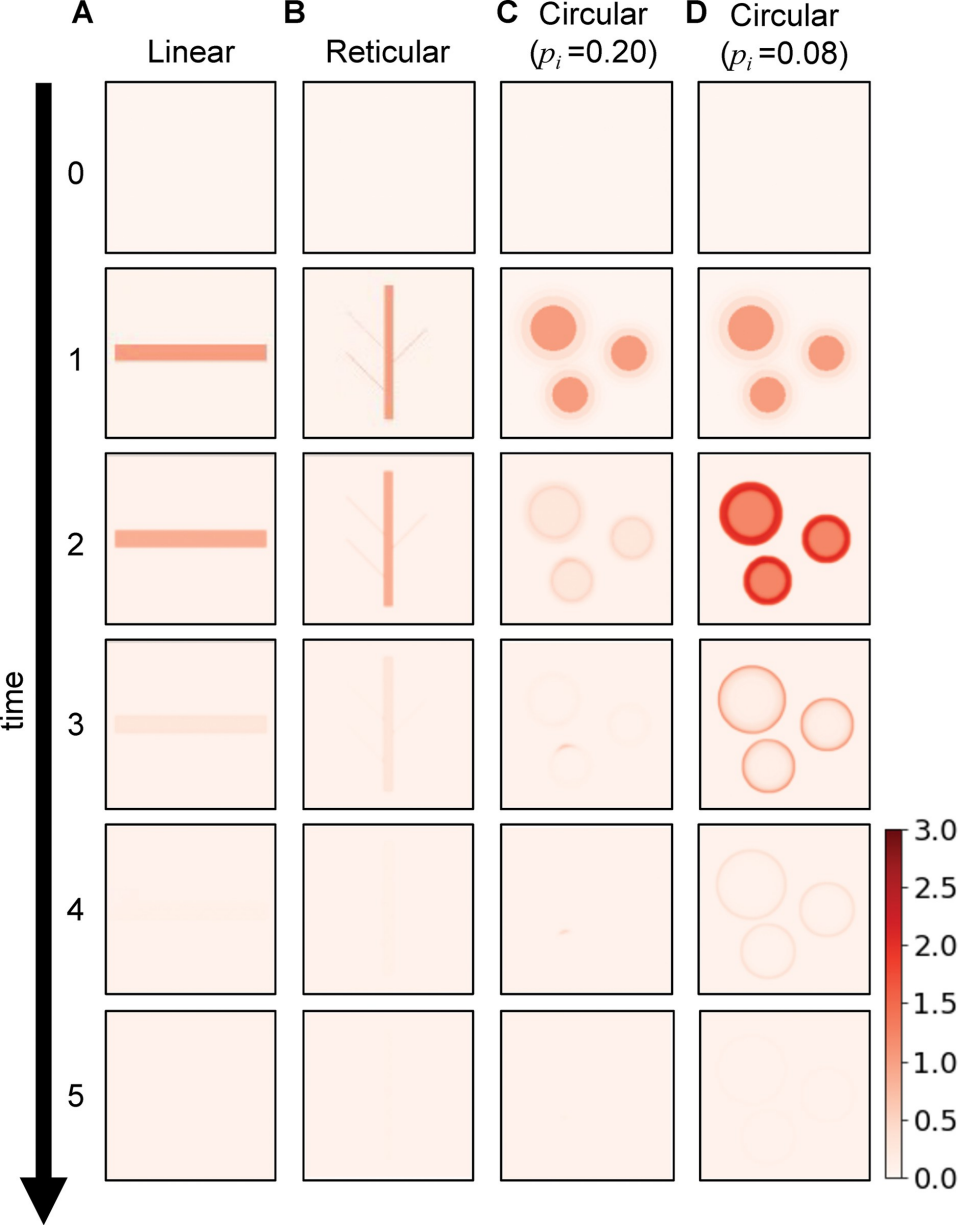
Simulated time courses of the healthy fading patterns. Spatiotemporal evolution of pro-inflammatory mediator levels (*a*) upon initial stimulation in linear **(A)**, reticular **(B)**, and circular areas **(C** and **D)**. The parameter values for these simulations are listed in S2(A)Table.

The development of the erythema pattern was simulated by numerically solving the initial value problem in Eq 2 using the classic Runge-Kutta method. The simulation was performed for cells aligned in a two-dimensional geometry with a periodic boundary condition (Fig 1J). As an initial condition of the simulation, pro- and anti-inflammatory mediators were uniformly set as 0.01 in the entire space (Fig 2, time = 0). Stimulation was introduced into these cells to induce erythema. For stimulation, we referred to the physiological condition at the onset of erythema, where a few small (~ 1 mm) inflamed areas exhibited a high concentration of pro-inflammatory mediators [5]. Accordingly, for each inflamed area, we set a circular area with a high concentration of pro-inflammatory mediators, given by a two-dimensional Gaussian distribution after some time steps (Fig 2C and 2D, time = 1). Given the initial conditions and stimulations, we investigated whether the reaction-diffusion model could reproduce erythema patterns. A simulation code written in C language is available from GitHub: https://github.com/MakiSudo/Erythema-Patterns/blob/main/AInondim.c

## Results

### Erythema patterns observed in eleven diseases

Previous disease-specific models have focused on multiple expanding patterns within a single disease, whereas few models have focused on studies reporting the same type of expanding pattern across different diseases [12]. Thus, we comprehensively examined the correspondence between diseases and expanding pattern types in terms of how many diseases commonly exhibit each pattern type and the number of types each disease exhibits. The collected photographs of erythema were categorized into the following five types based on the definitions of patterns published by the International League of Dermatological Societies [37]; circular pattern was characterized by a uniformly colored round pattern, annular pattern surrounded by a single ring, polycyclic pattern surrounded by multiple rings, arcuate pattern with a segmented ring, and gyrate pattern resembling wood-grain. We first examined the number of diseases that exhibited the same pattern type. Circular, annular, polycyclic, arcuate, and gyrate patterns were found in 7, 5, 4, 7, and 4 diseases, respectively (Table 2), thereby indicating that each of the five expanding patterns corresponded to multiple diseases. We examined the number of pattern types that appeared within a single disease. Consequently, eight diseases exhibited multiple pattern types across patients. For example, psoriasis exhibited all five pattern types and lupus erythematosus exhibited four (Table 2). The most frequently observed pair of pattern types in the same disease were annular and arcuate (four diseases: psoriasis, lupus erythematosus, bullous pemphigoid, and annular erythema), whereas the least frequent were circular and gyrate (one disease: psoriasis). These results indicated that each disease corresponded to multiple pattern types. Taken together, the correspondence between patterns and diseases is many-to-many rather than one-to-one, which suggests a unified spatiotemporal regulatory mechanism across diseases to form the five types of expanding patterns.

**Table 2:** Erythema patterns observed in eleven diseases. References for each case are listed in S1 Table [48–96].

Disease name	Circular	Annular	Polycyclic	Arcuate	Gyrate
Psoriasis	2	2	1	1	1
Lupus erythematosus	N/A	6	1	3	4
Bullous pemphigoid	2	3	N/A	3	N/A
Lyme disease	2	2	4	N/A	N/A
Erythema multiforme	3	N/A	8	1	N/A
Lymphoma	2	N/A	N/A	2	N/A
Annular erythema	N/A	4	N/A	2	N/A
Sjögren syndrome	N/A	N/A	N/A	2	1
Sweet’s syndrome	2	N/A	N/A	N/A	N/A
Nummular eczema	2	N/A	N/A	N/A	N/A
Erythema gyratum repens	N/A	N/A	N/A	N/A	1
Reported number of diseases	7	5	4	7	4

### Reaction-diffusion model reproduced the fading patterns

We then examined whether mediator production via feedback can generate and control the fading pattern, which remains uninvestigated in the reaction-diffusion models (Fig 1A, 1I and 1J; Eq 2 in Methods). Given the local stimulation reflecting the shape of animal tentacles or capillary structure [10,24,25], the present model reproduced a fading linear or reticular pattern, respectively (Fig 2A and 2B). With circular stimulation, the inflamed area decreased in redness without changing the diameter, and the interior of the inflamed area cleared first and eventually disappeared (Fig 2C). This result resembles the clinical situation of a fading circular pattern [19]. During the appearance of fading patterns, mediator levels transiently increased and then decreased to their original levels (S1A Fig), which is consistent with the excitatory time course of the normal inflammation model without mediator diffusion [14]. We further analyzed the parameters that controlled fading speed. The smaller the anti-inflammatory mediator’s basal secretion rate (*p*_*i*_), the slower the inflamed area disappeared (Fig 2D). Similar results were obtained when the production of pro-inflammatory mediators was high. These results demonstrate that regulatory feedback can generate a fading pattern in synergy with diffusion and control the fading speed.

### Reaction-diffusion model also reproduced diverse expanding patterns

**Fig 3:**
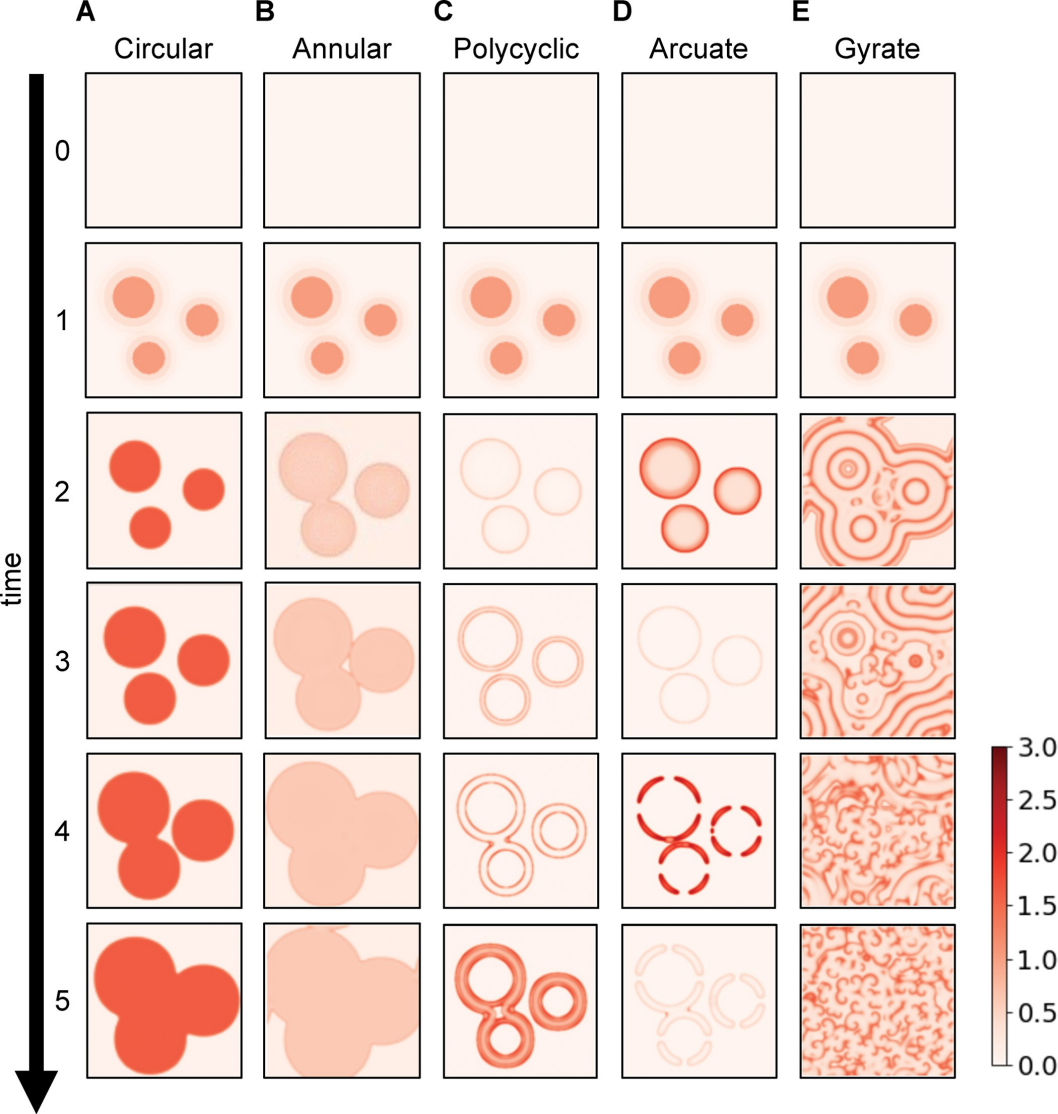
Simulated time courses of the five types of expanding patterns. Pro-inflammatory mediator levels (*a*). The initial stage of the inflamed area (row 1) consisted of three seed areas. Later forms of the disease (rows 2–5) correspond to circular **(A)**, annular **(B)**, polycyclic **(C)**, arcuate **(D),** or gyrate patterns **(E)**. The parameter values for these simulations are listed in S2(B) Table.

We examined whether any alteration in the model parameters (Table 1) could generate five expanding patterns. The model (Eq 2) showed that the inflamed area induced by transient local stimulation (Fig 3, time = 1) expanded centrifugally over time (Fig 3, time = 2–5). The inflamed area expanded with circular, annular, polycyclic, arcuate, or gyrate patterns, depending on the parameter values such as the degradation rate of the pro-inflammatory mediator (*r*_*a*_) or the anti-inflammatory mediator’s basal secretion rate (*p*_*i*_). The circular pattern appeared as round areas with a uniform concentration of pro-inflammatory mediators above a threshold (Fig 3A), thus, accounting for the uniformly colored round pattern in diseased skin [37]. The annular pattern showed areas with low pro-inflammatory mediator concentrations surrounded by a single boundary ring with higher concentrations (Fig 3B), which accounts for the inflamed areas surrounded by a single ring in diseased skin [37]. The polycyclic and arcuate patterns showed double concentric rings (Fig 3C) and segmented rings (Fig 3D), respectively. The gyrate pattern exhibited “C”-shaped double spirals resembling wood grains (Fig 3E). Moreover, the multiple expanding areas fused (Fig 3, time = 2–5), which was consistent with the clinical situation of expanding erythema [5]. Therefore, these simulated spatial patterns of pro-inflammatory mediators corresponded to each of the five types of expanding patterns in the clinical observations (Fig 1D–1H).

The inflammatory time course was further analyzed for each expanding pattern. When a circular pattern appeared, pro-inflammatory mediators maintained a persistently high concentration and failed to return to their original level (S1B Fig). When the annular pattern appeared, the mediator levels transiently increased and then decreased but did not return to their original levels (S1C Fig). These temporal dynamics are consistent with those of chronic inflammation [14]. In the case of the polycyclic, arcuate, and gyrate patterns, mediator concentrations transiently increased and then returned to their original levels, indicating excitability (S1D–S1F Fig). This result supports the presence of excitability in the development of gyrate patterns in erythema gyratum repens [16]. These results indicate that the alteration of the model parameters from the fading pattern can generate five types of expanding patterns.

### Transition to expanding patterns by alteration in the production of pro- and anti-inflammatory mediators

**Fig 4:**
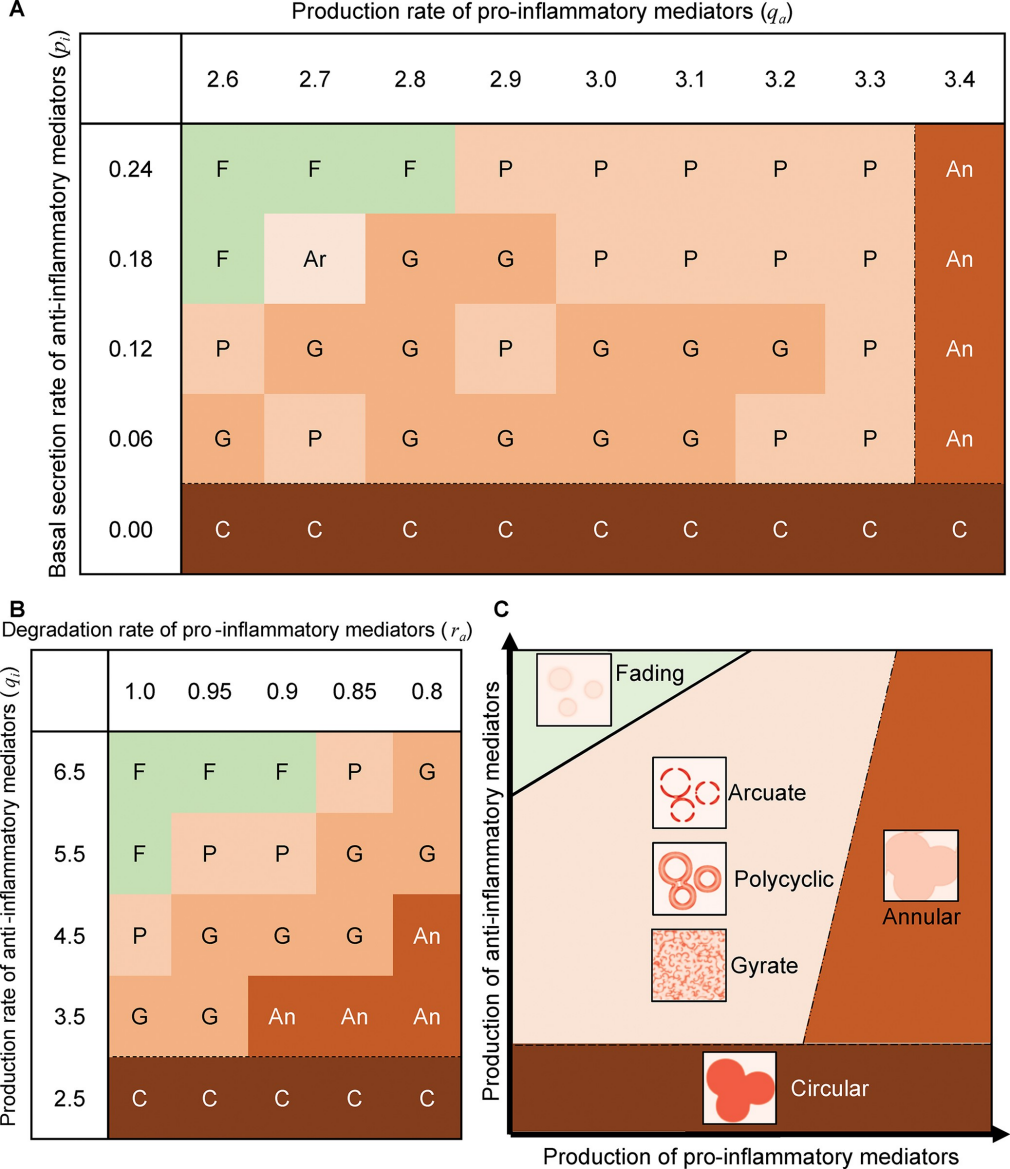
Pattern selection in the parameter space of pro- and anti-inflammatory mediator productions. Fading (F), arcuate (Ar), polycyclic (P), gyrate (G), annular (An) and circular (C) patterns emerged as the steady state (Eq 2) at the parameter values of *q*_*a*_ and *p*_*i*_ **(A)**, *r*_*a*_ and *q*_*i*_ **(B)**. *p*_*a*_ = 0.05, *r*_*a*_ = 0.8, *q*_*i*_ = 6.0 for (A) and *p*_*a*_ = 0.05, *q*_*a*_ = 3.0, *p*_*i*_ = 0.12 for (B). In all the simulations, *D*_*a*_ = *D*_*i*_ = 0.3. **(C)** Summary for all the analyzed parameter space regarding the mediator production (see also S2 and S3 Figs), indicating the characteristic imbalance by each expanding pattern.

To identify how the direction and severity of mediator production imbalance affects the pattern in the clinical spectrum of expanding patterns, we investigated the parameters (Table 1) affecting the transition from the fading pattern to each expanding pattern. First, increasing the pro-inflammatory mediator’s production rate (*q*_*a*_) from the parameter set of the fading pattern generated arcuate, gyrate, or polycyclic patterns (Fig 4A). A further increase in the pro-inflammatory mediator’s production rate (*q*_*a*_) brought about an annular pattern (Fig 4A). Conversely, with a decreasing basal secretion rate of the anti-inflammatory mediator (*p*_*i*_) from the parameter set of the fading pattern, arcuate, gyrate, polycyclic, and circular patterns appeared sequentially (Fig 4A). These transitions from the fading pattern to all five types of expanding patterns depending on *q*_*a*_ or *p*_*i*_ prompted us to hypothesize that increasing pro-inflammatory or decreasing anti-inflammatory mediator concentration can cause the transition from the fading pattern to transient expanding patterns (arcuate, gyrate, and polycyclic) and ultimately to chronic expanding patterns (annular and circular).

To test this hypothesis, we comprehensively investigated pattern transitions with alterations in each of the parameters affecting mediator concentration. Decreasing the degradation rate (*r*_*a*_) of the pro-inflammatory mediator and production rate of anti-inflammatory mediators (*q*_*i*_) from the fading pattern parameter set consistently led to polycyclic, gyrate, annular, and finally circular patterns (Fig 4B), thereby supporting this hypothesis. The results from various combinations of parameters identified the parameter regions for each expanding pattern in the clinical spectrum ranging from transient to chronic expanding patterns (Figs 4A, 4B, S2, and S3). The transient expanding pattern, including arcuate, gyrate, and polycyclic patterns, emerged under lower production of anti-inflammatory mediators and higher production of pro-inflammatory mediators compared to the fading pattern. Excessive imbalance resulted in a chronic expanding pattern; the annular pattern appeared under the overproduction of pro-inflammatory mediators, whereas the circular pattern appeared under the depletion of anti-inflammatory mediators. Generally, alterations in all parameters of feedback in the model caused an imbalance in mediator production, thereby resulting in transient and eventually chronic expanding patterns.

These results indicate the transition from each diseased expanding pattern to a healthy fading pattern. Specifically, the annular and circular patterns shifted to the fading pattern by reducing the production of pro-inflammatory mediators and increasing the production of anti-inflammatory mediators, respectively. Overall, these parameter-to-patterning correspondences showed that the two-dimensional space representing pro- and anti-inflammatory mediator production describes the clinical spectrum from the five types of expanding patterns in diseased skin to the fading pattern in healthy skin (Fig 4C).

### Stability of the healthy and inflamed states determines the expanding or fading patterns

**Fig 5:**
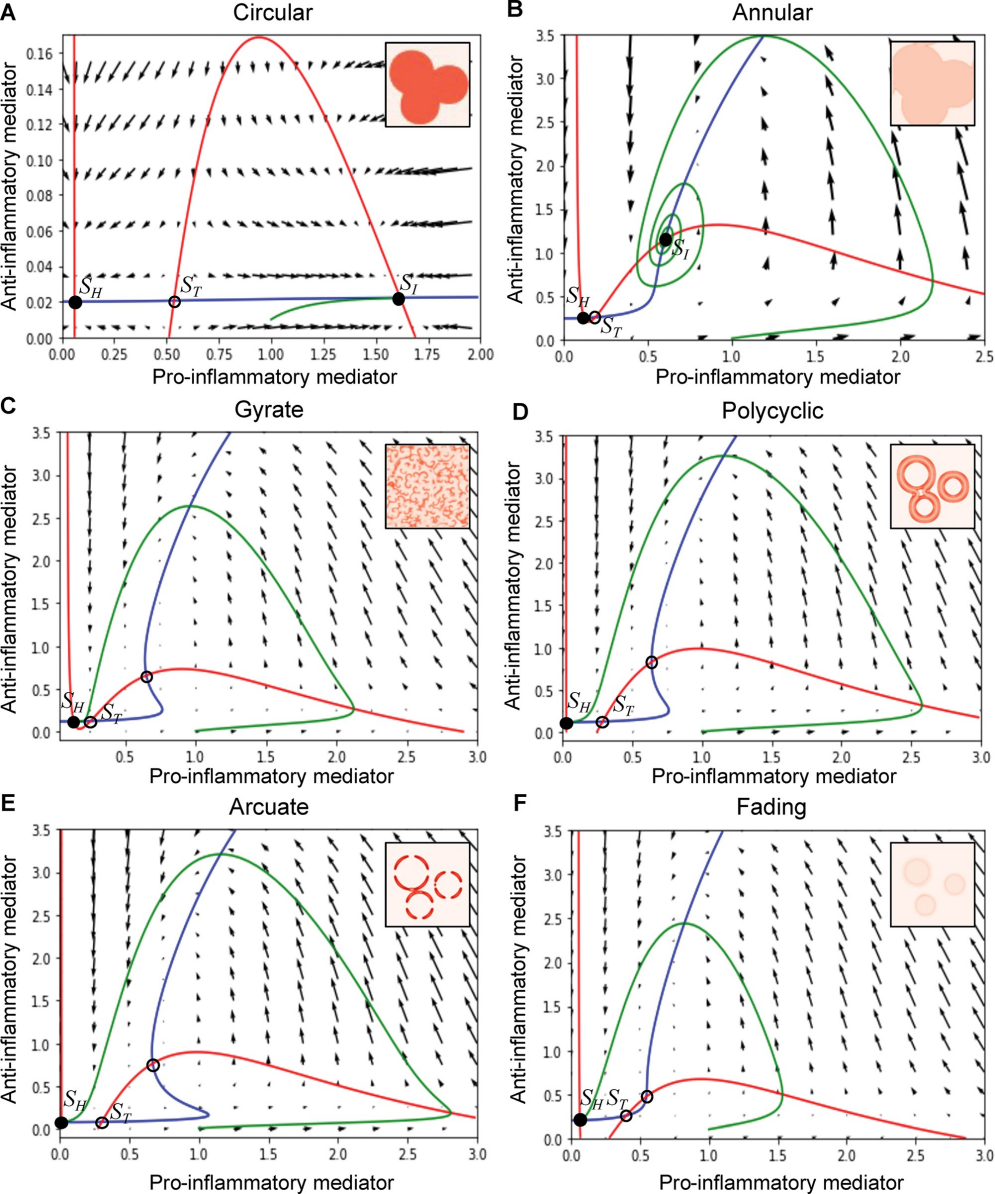
Dynamical characters underlying the five expanding and fading pattern types. The phase space of pro- and anti-inflammatory mediator concentrations (*a*, *i*) depicts the time course (green curve) upon stimulation and the nullclines (red curve for *da/dt* = 0 in Eq 2A; blue curve for *di/dt* = 0 in Eq 2B; *D*_*a*_ = *D*_*i*_ = 0). The intersections of the nullclines indicate steady states, where filled and hollow circles represent stable and unstable states, respectively. The time course shows convergence to a stable steady state upon a supra-threshold stimulation at the initial condition (*a* = 1.0, *i* =0.01). Vector fields are also shown to represent mediator dynamics at the respective concentration. The parameter values for each simulation are listed in S2 Table.

The number of stable states was analyzed to identify the dynamic properties underlying the differences between the fading pattern and each of the expanding patterns. These states were predicted as the temporal properties between normal and chronic inflammation, which are regulated by excitability and bistability, respectively [14], but remain unexamined regarding the spatial patterns. In the parameter set for the circular pattern, the regulatory feedback between pro- and anti-inflammatory mediators resulted in the bistability: two steady states are stable, given by low and high concentrations corresponding to the healthy (*S*_*H*_ in Fig 5A) and inflamed (*S*_*I*_ in Fig 5A) states, respectively, whereas there is an unstable steady state, corresponding to a threshold concentration (*S*_*T*_ in Fig 5A). Bistability also existed in the annular pattern (Fig 5B). For the circular and annular patterns, the concentrations of pro- and anti-inflammatory mediators eventually reached the inflamed state upon a suprathreshold stimulation (Fig 5A and 5B).

In contrast, in the gyrate, polycyclic, arcuate, and fading patterns, the regulatory feedback resulted in one stable and two unstable steady states, and the mediator concentrations eventually reached a healthy state (Fig 5C–5F). A major difference from the bistability exhibiting circular and annular patterns is the excitability, where the inflamed state is no more stable thereby only appearing in a transient manner upon stimulation. While the excitability and bistability underlie the fading and circular/annular patterns, respectively, consistently with the previous study [14], the present results further show that the excitability underlies some of the pathologic inflammation resulting in gyrate, polycyclic, and arcuate patterns as well.

The number of stable states determines the pattern regardless of the initial condition in the spatial distribution of mediator concentration. Similar to the fading pattern (Fig 2), the arcuate, polycyclic, and gyrate patterns with the excitability appeared reproducibly, independently of the initial conditions due to a single stable state *S*_*H*_ (Fig 5C–5F). Even in circular and annular patterns with bistability where the threshold *S*_*T*_ was closer to the inflamed state *S*_*I*_ than the healthy state *S*_*H*_ (Fig 5A and 5B), the final spatial pattern was dominated by the *S*_*I*_ independently of the initial condition. On the contrary, when *S*_*T*_ was closer to the *S*_*H*_ than the *S*_*I*_, the inflamed area shrank rather than fading (S4A Fig). These results are general outcomes of the traveling wave of bistable systems [36], and are consistent with the previous theoretical studies on inflammation [23,38].

Finally, we examined which differences of the steady states with excitability selectively result in gyrate, polycyclic, arcuate, or fading patterns. As a result, we found that the distance between the healthy state (*S*_*H*_) and the threshold state (*S*_*T*_, a closer unstable steady state to *S*_*H*_) was the smallest in the gyrate pattern and increased in the order of polycyclic, arcuate, slow fading pattern, and fast fading pattern (Figs 5C–5F and S4B, and S4C). The fast fading pattern showed a smaller trajectory (green curve in S4B and S4C Fig) of change in the mediator concentration than the slow fading pattern. Moreover, the larger distance between the healthy state and the threshold state represents higher stability of the healthy state against stimulations. These results indicate that the degree of stability of the healthy state, as well as the stability of the inflamed state, differs between erythema patterns. Therefore, erythema patterns on the skin surface reflect the dynamic balance in the stability of the healthy and inflamed states within the skin.

## Discussion

### Diffusive mediator feedback spatiotemporally regulates erythema patterns between healthy and diseased skin

The spatiotemporal regulation of inflammation is an important theme in biomedical research. Inflammation depends on the feedback of pro- and anti-inflammatory mediators; however, it remains unclear how the feedback regulates fading erythema in healthy skin and expanding erythema in diseased skin. Here, a reaction-diffusion model with mediator feedback (Fig 1) successfully reproduced the fading patterns (Fig 2) and five types of expanding patterns (Fig 3), thereby suggesting that feedback and diffusion can generate fading patterns in healthy skin and expanding patterns in eleven diseases (Table 2). The present study showed that parameter alterations in mediator production destabilized a stable steady state representing a healthy condition while in turn stabilizing the inflamed state (i.e., bifurcation from excitability to bistability [39]) and led to a transition from a fading pattern to five types of expanding patterns (Fig 5).

The parameter-to-patterning correspondence (Figs 4A, 4B, S2 and S3F) allows us to infer the pathogenesis mechanism in various diseases exhibiting each of diverse expanding patterns (seen in Table 2). For instance, psoriasis exhibits all five expanding patterns (Table 2) and increased levels of pro-inflammatory mediator (TNF-α) [19], which is consistent with our theoretical results. The elevated pro-inflammatory mediator in psoriatic skin has been suggested to be caused by genetic mutations affecting regulatory feedback [14]. Considering these previous studies, our model predicts a psoriasis progression where the fading pattern transits to arcuate, polycyclic, gyrate, annular, and circular patterns where an increase in the TNF-α level is possibly due to mutation-induced alteration in the feedback parameters, e.g., increase of the production of pro-inflammatory mediator *q*_*a*_ (Fig 4A). Alternatively, lyme disease exhibits circular, annular, and polycyclic patterns (Table 2). A clinical report showed that patients in Missouri predominantly exhibit an annular pattern without prognostic symptoms, while those in New York tend to exhibit a circular pattern with prognostic symptoms following the same treatment [13]. Considering our theoretical result that the overproduction of pro-inflammatory mediators and the depletion of anti-inflammatory mediators leads to the annular and circular pattern, respectively (Figs 4, 5A and 5B), altered levels of pro-inflammatory and anti-inflammatory mediators may significantly impact the development and prognosis of lyme disease in Missouri and New York patients, respectively.

These qualitative parameter estimations will be verified in the future through parameter quantification in each diseased skin exhibiting any expanding patterns. By incorporating this quantitative correspondence between patterns and parameters measured in each disease into the present model, we would develop each disease-specific model with a quantitative predictability of how much change of the skin parameters transit from healthy to diseased pattern or vice versa. Therefore, this study provides the first step to controlling the health-to-disease transition of skin inflammation via diffusive mediator feedback.

### Prospective treatment from the model prediction

Mediator feedback parameter-dependent transitions from each expanding to fading pattern (Figs 4, S2 and S3) suggest effective treatment strategies depending on skin barrier conditions. Experimental findings demonstrated that the maximum production rate (*Q*_*A*_ in Eq 1A and *q*_*a*_ in Eq 2A) and basal secretion rate (*P*_*A*_ in Eq 1B and *p*_*a*_ in Eq 2B) of pro-inflammatory mediators are significantly lower in healthy skin than in diseased skin with a deterioration of the skin microbiome [7,40,41] and in diseased skin with defects in the integrity of physical barriers, respectively [6,42].

Observation of erythema patterns under different skin barrier conditions reveals the influence of skin barrier conditions on the model parameters and thus, provides potential treatments to reduce the maximum production rate or the basal secretion rate of pro-inflammatory mediators. For example, probiotics, which improve the composition of the skin microbiome, significantly reduce the maximum production rate of pro-inflammatory mediators [43,44]. Additionally, probiotics can improve the integrity of physical barriers [43], thus, reducing the basal secretion rate of pro-inflammatory mediators. Therefore, probiotics can be a prospective treatment leading to a fading pattern. Further experimental studies on the influence of skin barrier conditions on erythema patterns will offer deeper insights into the development of effective treatments for erythema associated with inflammatory skin disease.

### Applicability of the present model

This study provides a systematic definition of disease severity using this model. The model describes the expanding patterns and fading patterns on the same parameter space (Fig 4), which represents how far each expanding pattern is from the fading pattern. This distance is similar to the state-space representation of inflammatory responses, where disease severity is measured as the distance between a patient’s coordinates and that of one of the disease states [45,46]. Defining disease severity as the distance between the fading pattern and erythema patterns on the patient’s skin will help estimate the appropriate dosage and strength of treatment for each patient based on their erythema pattern.

Our framework can also predict the disease risk in healthy individuals. The model showed that the fading patterns disappeared at different speeds depending on the parameters (Fig 2C and 2D). This means that the parameters in healthy individuals can be estimated by measuring fading speeds using patch tests. Utilizing the obtained parameters, the disease risk of each individual can be evaluated as the distance from the parameter that shows the expanding patterns. Therefore, we propose that the future integration of models, experimental findings, and clinical data will allow for the development of personalized treatment and prediction of inflammatory skin diseases in a noninvasive manner.

### Future implications

Although this study showed the reaction-diffusion model for the fading pattern and five expanding patterns, there are two major limitations. Firstly, the expanding patterns continued to expand in the present model simulations (Fig 3), while the actual erythema typically stopped expanding and maintained its size in the clinical observation [5,20]. This is probably because the present model focuses on the non-chemotactic cells (e.g., including keratinocytes), whereas chemotactic cells (e.g., macrophages and neutrophils) also contribute to skin inflammation [2,3]. Moreover, the present model focuses on the innate immune response, whereas the skin initiates an acquired immune response in the persistence of the innate immune response. Therefore, incorporating the chemotactic cells and acquired immune response into the model will reproduce the end of the expansion.

Secondly, we focused on well-circumscribed erythema with clear boundaries (Fig 3) that resulted from inflammation in the upper layers of the skin. Inflammation in the deeper layers of the skin leads to poorly circumscribed erythema with a gradual transition between the affected area and healthy skin [47]. Future studies incorporating the three-dimensional structure of the skin into the present model would take into account poorly circumscribed erythema.

## Conclusions

Here, positive and negative feedback and diffusion of pro- and anti-inflammatory mediators were demonstrated to commonly account for the fading patterns in healthy skin and five types of expanding patterns in diseased skin. Mechanistically, alterations in mediator production destabilize a healthy state while stabilizing an inflamed state, thereby resulting in a transition to diverse expanding patterns. The mediator feedback dynamics is the fundamental regulator of the health-to-disease transition, which suggests effective treatment strategies for each expanding pattern. Therefore, regulating mediator production provides an experimentally testable framework for the spatiotemporal regulation of erythema, which can facilitate the development of a noninvasive and personalized treatment for inflammatory skin diseases.

## Supporting information

S1 Table List of references for erythema observed in the eleven diseases. (XLSX)

S2 Table Parameter values used in the simulations. Parameter values used to generate the fading patterns in Fig 2 (A) and the five types of expanding patterns in Fig 3 (B).
(XLSX)

S1 Fig Temporal evolution of mediator concentrations in the fading or expanding patterns. Blue and red lines represent the concentrations of the pro- and anti-inflammatory mediators, respectively. A high concentration of pro-inflammatory mediator was transiently applied at time = 10 to 11. *D*_*a*_ = *D*_*i*_ = 0; and the other parameter values for these simulations are listed in S2 Table.
(TIF)

S2 Fig Pattern selection in the parameter space of pro- and anti-inflammatory mediator productions. Fading (F), arcuate (Ar), polycyclic (P), gyrate (G), annular (An) and circular (C) patterns emerged as the steady state (Eq 2) at the parameter values of *q*_*a*_ and *r*_*a*_ (**A**), *q*_*a*_ and *q*_*i*_ (**B**), *p*_*a*_ and *p*_*i*_ (**C**), *q*_*a*_ and *p*_*a*_ (**D**). Simulations of the gray areas did not correspond to any of the five patterns. *p*_*a*_ = 0.02, *p*_*i*_ = 0.12, *q*_*i*_ = 6.0 for (**A**), *p*_*a*_ = 0.02, *r*_*a*_ = 0.8, *p*_*i*_ = 0.12 for (**B**), *q*_*a*_ = 3.0, *r*_*a*_ = 0.8, *q*_*i*_ = 6.0 for (**C**), *r*_*a*_ = 0.8, *p*_*i*_ = 0.12, *q*_*i*_ = 6.0 for (**D**). In all simulations, *D*_*a*_ = *D*_*i*_ = 0.3.
(TIF)

S3 Fig Alterations in the production rates of pro- and anti-inflammatory mediators transition from fading patterns to various expanding patterns. Representation of the patterns generated using Eq 2 for different values of the parameters *r*_*a*_ and *p*_*a*_ (**A**), *r*_*a*_ and *p*_*i*_ (**B**), *p*_*a*_ and *q*_*i*_ (**C**), and *p*_*i*_ and *q*_*i*_ (**D**). Simulations of the gray areas did not correspond to any of the five patterns. *q*_*a*_ = 3.0, *p*_*i*_ = 0.12, *q*_*i*_ = 6.0 for (**A**), *p*_*a*_ = 0.05, *q*_*a*_ = 3.0, *q*_*i*_ = 6.0 for (**B**), *q*_*a*_ = 3.0, *r*_*a*_ = 0.95, *p*_*i*_ = 0.12 for (**C**), and *p*_*a*_ = 0.05, *q*_*a*_ = 3.0, *r*_*a*_ = 0.95 for (**D**). In all the simulations, *D*_*a*_ = *D*_*i*_ = 0.3.
(TIF)

S4 Fig Simulated time courses of shrinkage pattern and dynamical characters underlying the fading pattern. (**A)** Simulated time courses of shrinkage pattern. *p*_*a*_ = 0.03, *q*_*a*_ = 2.0, *r*_*a*_ = 0.99, *D*_*a*_ = 0.3, *p*_*i*_ = 0.02, *q*_*i*_ = 6.0, *D*_*i*_ = 0.3. (**B)** and (**C)** Dynamical characters underlying the fast (**B**) and slow (**C)** fading pattern. (**B**) is the same as Fig 5F.
(TIF)
