# Supplementary figures and images for: Diffusive mediator feedbacks control the health-to-disease transition of skin inflammation
Source: PLoS Comput Biol. 2024 Jan 18;20(1):e1011693. doi: 10.1371/journal.pcbi.1011693 (PMC10796066; doi:10.1371/journal.pcbi.1011693)

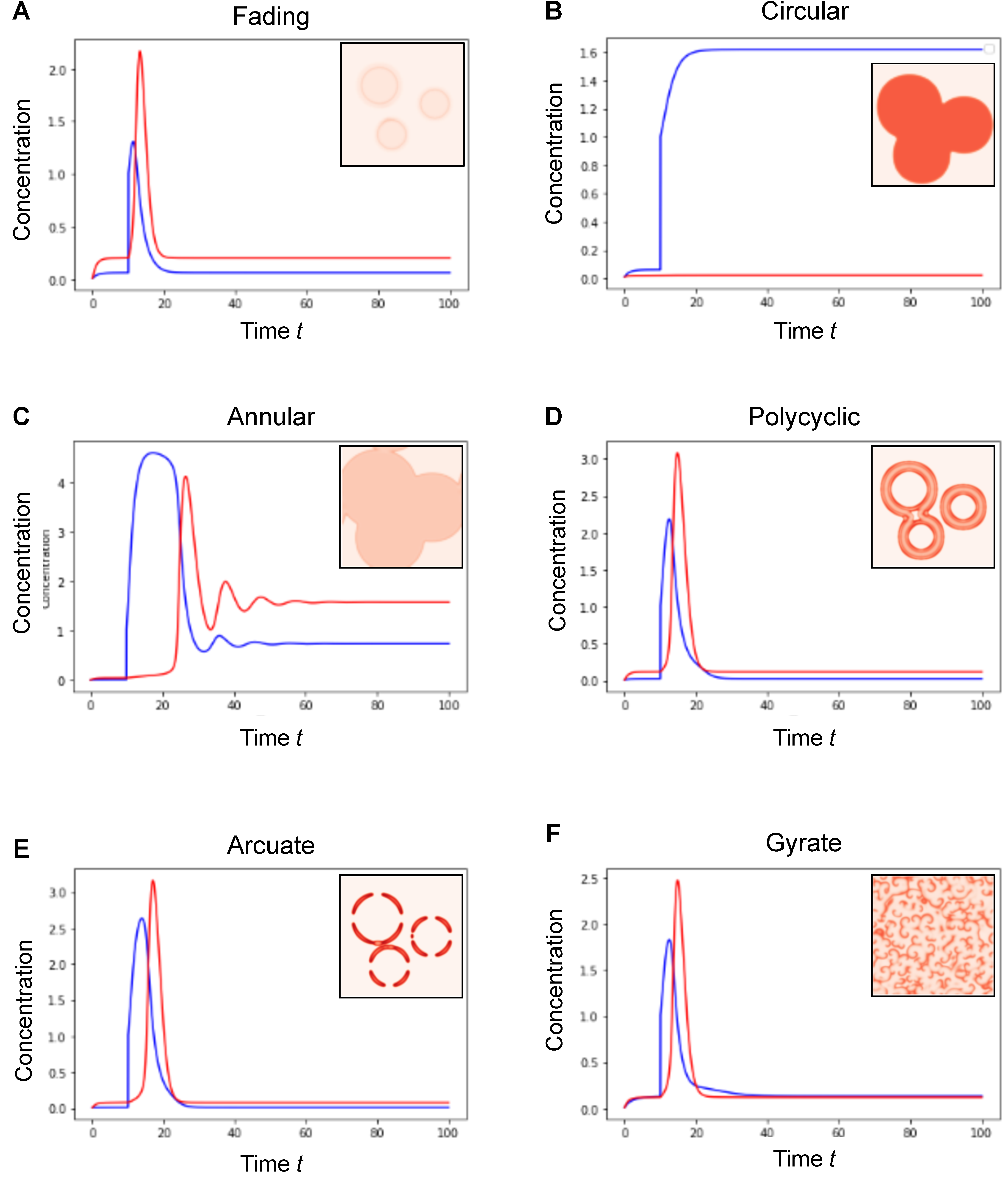

Supplement: S1 Fig — Blue and red lines represent the concentrations of the pro- and anti-inflammatory mediators, respectively. A high concentration of pro-inflammatory mediator was transiently applied at time = 10 to 11. Da = Di = 0; and the other parameter values for these simulations are listed in S2 Table. (TIF) [file pcbi.1011693.s003.tif]

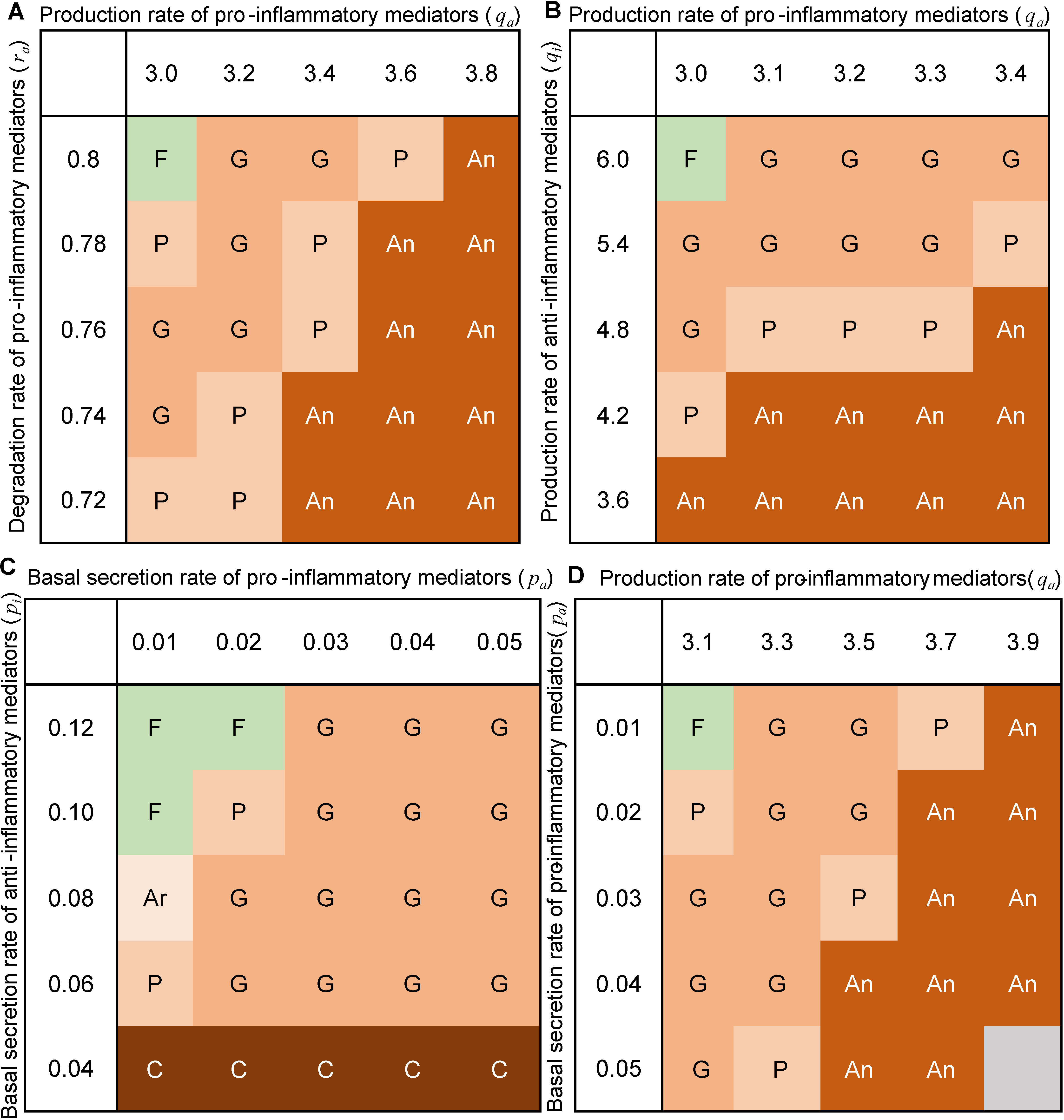

Supplement: S2 Fig — Fading (F), arcuate (Ar), polycyclic (P), gyrate (G), annular (An) and circular (C) patterns emerged as the steady state (Eq 2) at the parameter values of qa and ra (A), qa and qi (B), pa and pi (C), qa and pa (D). Simulations of the gray areas did not correspond to any of the five patterns. pa = 0.02, pi = 0.12, qi = 6.0 for (A), pa = 0.02, ra = 0.8, pi = 0.12 for (B), qa = 3.0, ra = 0.8, qi = 6.0 for (C), ra = 0.8, pi = 0.12, qi = 6.0 for (D). In all simulations, Da = Di = 0.3. (TIF) [file pcbi.1011693.s004.tif]

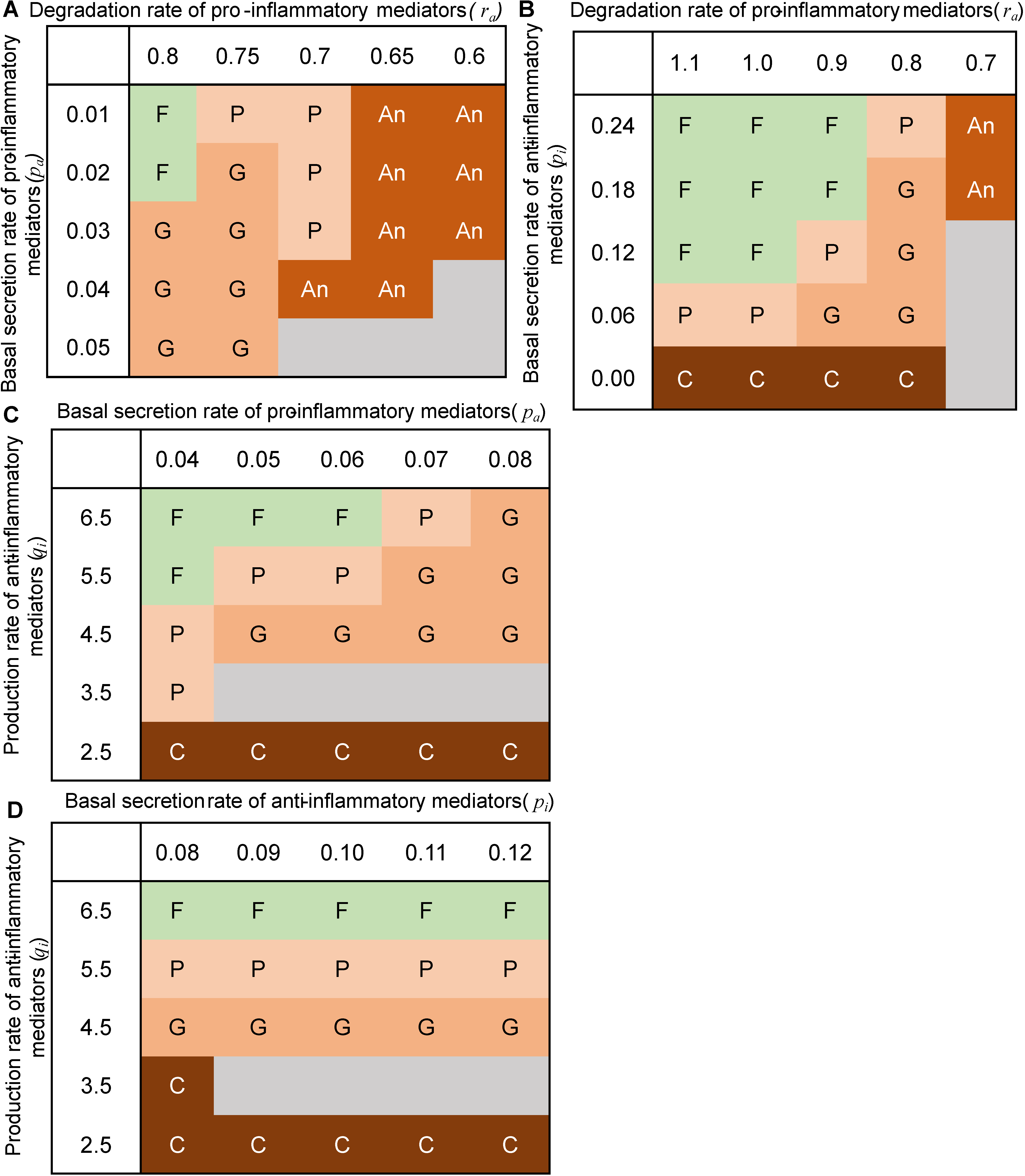

Supplement: S3 Fig — Representation of the patterns generated using Eq 2 for different values of the parameters ra and pa (A), ra and pi (B), pa and qi (C), and pi and qi (D). Simulations of the gray areas did not correspond to any of the five patterns. qa = 3.0, pi = 0.12, qi = 6.0 for (A), pa = 0.05, qa = 3.0, qi = 6.0 for (B), qa = 3.0, ra = 0.95, pi = 0.12 for (C), and pa = 0.05, qa = 3.0, ra = 0.95 for (D). In all the simulations, Da = Di = 0.3. (TIF) [file pcbi.1011693.s005.tif]

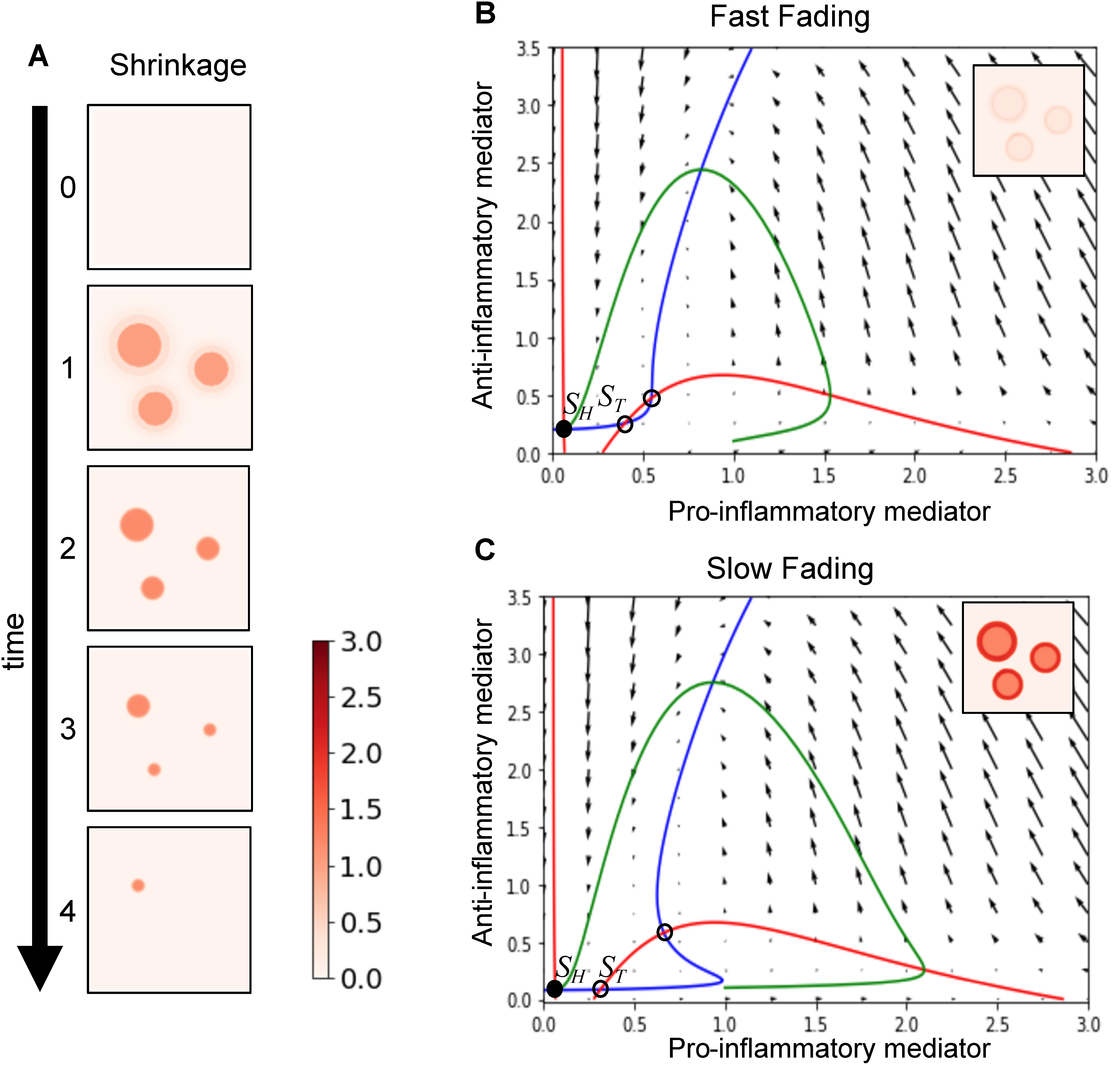

Supplement: S4 Fig — (A) Simulated time courses of shrinkage pattern. pa = 0.03, qa = 2.0, ra = 0.99, Da = 0.3, pi = 0.02, qi = 6.0, Di = 0.3. (B) and (C) Dynamical characters underlying the fast (B) and slow (C) fading pattern. (B) is the same as Fig 5F. (TIF) [file pcbi.1011693.s006.tif]
