# Supplementary material for: Diffusive mediator feedbacks control the health-to-disease transition of skin inflammation
Source: PLoS Comput Biol. 2024 Jan 18;20(1):e1011693. doi: 10.1371/journal.pcbi.1011693 (PMC10796066; doi:10.1371/journal.pcbi.1011693)
Supplement: Attachment — Submitted filename: 137178_1_rebuttal_2848231_s3y9rg.pdf [file pcbi.1011693.s007.pdf]

Full Revision

Manuscript number: RC-2023-02034

Corresponding author(s): Maki, Sudo

1. General Statements [optional]

Thank you for inviting us to submit our revised manuscript titled, “Diffusive mediator feedbacks control the health-to-disease transition of skin inflammation.” We appreciate the time and effort the editor and each of the reviewers have dedicated to providing insightful feedback on ways to strengthen our manuscript. The revisions in the main text in response to the detailed comments are highlighted in red and were proofread by professional English editors. We hope that our revision and responses address all the concerns raised by the reviewer, and we look forward to hearing from you regarding this submission.

2. Point-by-point description of the revisions

This section is mandatory. Please insert a point-by-point reply describing the revisions that were already carried out and included in the transferred manuscript.

Reviewer #1 (Evidence, reproducibility and clarity (Required)):

>The manuscript provides a model of interacting populations of pro- and anti-inflammatory mediators to explain spatial patterns associated with various inflammatory conditions. The work is robust and articulated well, and is certainly scientifically relevant.

Authors: Thank you for your positive evaluation and many insightful comments on our manuscript. We have incorporated your feedback, and hope that our revisions satisfy all the comments.

>Minor amendments:

Personally, I feel that the model should be reported prior to the results, as the choice of model is likely to have great significance on the observations. It would be preferable for the reader to have a clear picture of the governing equations in their mind as they digest the results.

Au: Following this reviewer's suggestion, we have relocated the Method section including the model description to be written prior to the Result section (p.9-14 lines 152-232; revised manuscript).

>The literature review is largely relatively thorough; however, I think it is important that the previous works of Joanne Dunster (University of Reading) and collaborators are included, as these are very closely related to this work. In particular, the authors should note the following two papers, which take a spatial approach:

- Bayani, A., Dunster, J.L., Crofts, J.J. et al. Mechanisms and Points of Control in the Spread of Inflammation: A Mathematical Investigation. Bull Math Biol 82, 45 (2020). https://doi.org/10.1007/s11538-020-00709-y
- Bayani A, Dunster JL, Crofts JJ, Nelson MR (2020) Spatial considerations in the resolution of inflammation: Elucidating leukocyte interactions via an experimentally-calibrated agent-based model. PLoS Comput Biol 16(11): e1008413. https://doi.org/10.1371/journal.pcbi.1008413

Au: We have incorporated this comment by adding the two suggested papers to the relevant sentences in the literature review (p.6 line 118-119; revised manuscript) as follows: “Previous reaction-diffusion models, including chemotactic cells, have reproduced the resolution of inflammation in the lung [Bayani et al. 2020a, Bayani et al. 2020b]”

>One key point that should be mentioned in the discussion is that the model neglects any immune cells (e.g. neutrophils, macrophages) which contribute greatly to the inflammatory condition. Since these cells are motile, and also can contribute both pro- and anti-inflammatory effects, they are likely to influence spatial patterns significantly. It is not necessarily a problem that these aren't included in the model, but I feel that it is important that their omission be discussed in the manuscript.

Au: We have now discussed the immune cells in the “Future implications” as the reviewer suggested (p.29 line 477-483; revised manuscript) as follows: “This is probably because the present model focuses on the non-chemotactic cells (e.g., including keratinocytes), whereas chemotactic cells (e.g., macrophages and neutrophils) also contribute to skin inflammation [Zhang and An 2007, Coondoo 2011]. Moreover, the present model focuses on the innate immune response, whereas the skin initiates an acquired immune response in the persistence of the innate immune response. Therefore, incorporating the chemotactic cells and acquired immune response into the model will reproduce the end of the expansion.”

Reviewer #1 (Significance (Required)):

The manuscript advances our current understanding of spatially spreading inflammation and corresponding patterns, but needs to be contextualized against existing literature as described above. This manuscript will appeal to theoreticians (Mathematicians) and clinicians/experimentalists alike.

Reviewer #2 (Evidence, reproducibility and clarity (Required)):

>The authors propose a minimal mechanistic mathematical model able to reproduce qualitatively different spatial patterns observed in healthy and disease epidermis. The starting point is a systematic review of medical images of different dermatological conditions, which they classify and successfully capture according to the spatial patterns. It is an interesting piece of work, but I consider that it will gain significance if the theoretical results are compared again with the clinical data. Specifically, the authors show a very interesting map between parameter regions and different spatial patterns; this result should be compared back to clinical data, to confirm that specific changes in spatial patterns indeed result from predicted changes in a specific parameter (e.g., due to a genetic condition that affects a feedback strength).

Authors: We thank you for providing your valuable comments on our manuscript.

Following your suggestion about the comparison of theoretical results with the clinical data, we have predicted which specific parameters including the feedback strength cause specific transitions of

spatial patterns in the respective diseases. The discussion was added on p.26 lines 415-438 in the revised manuscript as follows: “The parameter-to-patterning correspondence (Fig. 4A, B, S2 Fig., and S3 Fig.) allows us to infer the pathogenesis mechanism in various diseases exhibiting each of diverse expanding patterns (seen in Table 2). For instance, psoriasis exhibits all five expanding patterns (Table 2) and increased levels of pro-inflammatory mediator (TNF-α) [Ringham et al. 2019], which is consistent with our theoretical results. The elevated pro-inflammatory mediator in psoriatic skin has been suggested to be caused by genetic mutations affecting regulatory feedback [Valeyev et al. 2010]. Considering these previous studies, our model predicts a psoriasis progression where fading pattern transits to arcuate, polycyclic, gyrate, annular, and circular pattern where increase in the TNF-α level is possibly due to mutation-induced alteration in the feedback parameters, e.g., increase of the production of pro-inflammatory mediator qa (Fig. 4A). Alternatively, Lyme disease exhibits circular, annular, and polycyclic patterns (Table 2). A clinical report showed that patients in Missouri predominantly exhibit an annular pattern without prognostic symptoms, while those in New York tend to exhibit a circular pattern with prognostic symptoms following the same treatment [Wormser et al. 2005]. Considering our theoretical result that the overproduction of pro-inflammatory mediators and the depletion of anti-inflammatory mediators leads to the annular and circular pattern, respectively (Fig 4, 5A, and B), altered levels of pro-inflammatory and anti-inflammatory mediators may significantly impact the development and prognosis of Lyme disease in Missouri and New York patients, respectively.

These qualitative parameter estimations will be verified in the future through parameter quantification in each diseased skin exhibiting any expanding patterns. By incorporating this quantitative correspondence between patterns and parameters measured in each disease into the present model, we would develop each disease-specific model with a quantitative predictability of how much change of the skin parameters transit from healthy to diseased pattern or vice versa. Therefore, this study provides the first step to controlling the healthy-to-diseased transition of skin inflammation via diffusive mediator feedback.”

>Another shortcoming of this work is that some of the conclusions are rushed: the parameter-to-spatial patterns analysis would strongly benefit from adding a quantitative to the qualitative description, e.g., mapping how changes in a given parameter value results in gradual changes in fading speed. Along the same line, the stability analysis for the different fading pattens was performed only for selected parameter values, it is not clear how variations in parameter values affect the sizes of the basins of attraction of the different steady states; we want to make sure that the parameter values were not cherry-picked. Further, given that the authors show bistability for some parameter values, then the dependency on initial conditions on the final spatial pattern should be more extensively investigated.

Au: We have incorporated these comments by adding a quantitative description including new results and future research strategies following each of the three constructive suggestions raised by the reviewer.

First, regarding “the fading speed” the reviewer suggested, fading speed is affected by changes in parameters involved in mediator production. In particular, the speed is reduced by an increase in the production parameters of pro-inflammatory mediators (p_a, q_a) and a decrease in those of anti-inflammatory mediators (p_i, q_i) (Fig.2. C and D). Moreover, “the size of the basins” the reviewer pointed out corresponds to the distance between S_T (Threshold) and S_H (Healthy state) in the cases with excitability. The distance between S_T and S_H becomes closer indicating the health state being less stable when pro-inflammatory mediators (p_a, q_a) increase or anti-inflammatory mediators (p_i, q_i) decrease from the healthy fading pattern. The imbalance of the mediator production transits the fast fading pattern with a small trajectory into a slow fading pattern with a larger trajectory. As imbalance goes on, the expanding pattern appears in the order of arcuate, polycyclic, and gyrate (Fig. 5). In cases with bistability, the size of basins corresponds to the relative distance S_T to S_H and S_T to S_I (Inflamed state). The circular and annular patterns appear when the distance between S_T and S_H is closer. On the other hand, when the distance between S_T and S_I was closer, the inflamed area shrank rather than expanded. The shrinking pattern appeared by reducing the production of pro-inflammatory mediators (p_a, q_a) or increasing the production of anti-inflammatory mediators (p_i, q_i) under conditions of stability. We have added a new figure and

described this finding in Results (p.24 lines 384-388; revised manuscript) as follows: “As a result, we found that the distance between the healthy state (S_H) and the threshold state (S_T, a closer unstable steady state to S_H) was the smallest in the gyrate pattern and increased in the order of polycyclic, arcuate, slow fading pattern, and fast fading pattern (Fig. 5C–F, S4 Fig. B and C). The fast fading pattern showed a smaller trajectory (green curve in S4 Fig. B and C) of change in the mediator concentration than the slow fading pattern.”

Second, regarding “the dependency on initial conditions”, we have further added a new result (p.24 line 374-382; revised manuscript) as follows: “The number of stable states determines the pattern regardless of the initial condition in the spatial distribution of mediator concentration. Similar to the fading pattern (Fig. 2), the arcuate, polycyclic, and gyrate patterns with the excitability appeared reproducibly, independently of the initial conditions due to a single stable state S_H (Fig. 5C-F). Even in circular and annular patterns with bistability where the threshold S_T was closer to the inflamed state S_I than the healthy state S_H (Fig. 5A-B), the final spatial pattern was dominated by the S_I independently of the initial condition. On the contrary, when S_T was closer to the S_H than the S_I, the inflamed area shrank rather than fading (S4 Fig. A). These results are general outcomes of the traveling wave of bistable systems [Murray 2002], and consistent with the previous theoretical studies on inflammations [Sudo and Fujimoto 2022, Volpert 2009]. ”

Finally, we have added “a quantitative to the qualitative description as a future research strategy (p.27 line 432-438; revised manuscript) as follows: “These qualitative parameter estimations will be verified in the future through parameter quantification in each diseased skin exhibiting any expanding patterns. By incorporating this quantitative correspondence between patterns and parameters measured in each disease into the present model, we would develop each disease-specific model with a quantitative predictability of how much change of the skin parameters transit from healthy to diseased pattern or vice versa. Therefore, this study provides the first step to controlling the healthy-to-diseased transition of skin inflammation via diffusive mediator feedback.”

>For reproducibility it is essential that the authors add a much more detailed description of the methods, including the software tools / numerical analysis tools used. Making the code publicly available would also be very beneficial to ensure the reproducibility of the results.

Au: Following your suggestion, we have added a description of the methods, including the simulation code, to the “Methods” (p.13 lines 231-232; revised manuscript) as follows: “A simulation code written in C language is available from GitHub: https://github.com/MakiSudo/Erythema-Patterns/blob/main/AInondim.c.”

>In conclusion, the work is very interesting and worth publishing, but requires (a) to come back to the clinical data for validation of model predictions, (b) a more thorough and quantitative investigation of the effects of parameter variations on model behaviors, (c) a more rigorous and systematic presentation of the methods, (d) carefully explaining how the proposed model is similar / differs to the classical activator - inhibitor model proposed by Turing, and (e) discussing / showing if the fading patterns result from a turning instability.

Au: For (a) “validation of model predictions,” (b) “model behaviors,” and (c) “a more rigorous and systematic presentation of the methods,” we have reflected your suggestions in the revised manuscript as described above.

Regarding (d) and (e), we have added an explanation of “how the proposed model is similar/differs to the classical activator–inhibitor model” and “if the fading patterns result from Turing instability” after the model construction in Methods (p.11-12 line 210-216; revised manuscript) as follows: “Reaction terms of this model are similar to the classical activator-inhibitor model proposed by Turing [Turing 1952], which includes the negative feedback of the activator through the inhibitor and the positive feedback of the activator. These reaction terms potentially result in Turing instability. However, the present model setting does not show Turing instability. The reason is that Turing instability requires a large difference between the diffusion coefficients of the activator and inhibitor [Murray 2002], whereas

these coefficients in the present model were set to be equal based on molecular findings that these molecular weights are close in proximity [Coondoo 2011]. ”

Referees cross-commenting

I agree with the comments from Reviewer #1.

Reviewer #2 (Significance (Required)):

The work aims to bridge mathematical modelling to dermatological practice, which is much needed to enable the use of theoretical and computational tools to clinical decision-making. While some mathematical models of skin inflammation have been proposed in the past (refer to papers from the RJ Tanaka group in systems dermatology), most of these do not consider explicitly the spatial component, which is crucial for modelling the clinically visible spatial patterns. Potentially interested audience includes biomathematicians, systems biologists, systems dermatologists, and, if the validation of the model predictions is achieved (as suggested above), also dermatologists.

I am a systems biologists working on multi-scale mechanistic mathematical modelling of epithelial tissue diseases. The work I just reviewed falls exactly within my area of expertise.
